# Minimally Invasive Versus Conventional Colectomy: Evaluating Clinical Outcomes, Complications, and Recovery in Modern Surgical Practice

**DOI:** 10.7759/cureus.99099

**Published:** 2025-12-13

**Authors:** Vaseem Akram Vadhooth, Krishnaprasad K, Priyanka L Reddy, Sailesh Kumar S

**Affiliations:** 1 Department of General Surgery, Sri Devaraj Urs Academy of Higher Education and Research, Kolar, IND

**Keywords:** colorectal surgery, enhanced recovery, minimally invasive colectomy, postoperative outcomes, surgical innovation

## Abstract

Minimally invasive colectomy (MIC) has transformed colorectal surgery by improving recovery, reducing morbidity, and enhancing postoperative quality of life, yet variations in clinical outcomes, learning curves, and cost-effectiveness continue to challenge universal adoption. This systematic review synthesized current evidence comparing MIC and open colectomy (OC) across clinical, functional, and economic outcomes, following PRISMA 2020 guidelines. A structured search of PubMed, Embase, Scopus, Web of Science, and CENTRAL identified randomized controlled trials, multicenter cohorts, and meta-analyses published between 2015 and 2025. Studies were included only if they compared MIC and OC in adults (≥18 years) and reported extractable quantitative outcomes. Across eligible studies, MIC demonstrated a mean reduction in hospital length of stay (LOS) of approximately two to three days, an effect size ranging from 0.45 to 0.62 for postoperative morbidity reduction, and a 40-60% decrease in intraoperative blood loss, while maintaining comparable oncologic parameters to OC. Integration with Enhanced Recovery After Surgery (ERAS) protocols further improved bowel recovery, mobilization, and discharge timelines without increasing complications. Risk-of-bias assessments using Cochrane RoB 2.0 and the Newcastle-Ottawa Scale indicated predominantly low-risk evidence, strengthening confidence in the findings. Inclusion of elderly and emergency populations demonstrated that MIC remains safe and reproducible across complex settings. Although robotic colectomy increases operative time and cost, these drawbacks are offset by accelerated recovery and reduced readmissions. Collectively, the quantitatively reinforced evidence supports the growing role of MIC, particularly when combined with ERAS principles, as an efficient and patient-centered approach in modern colorectal practice.

## Introduction and background

Colectomy remains a fundamental surgical intervention for a wide range of benign and malignant colonic diseases, including colorectal cancer, inflammatory bowel disease, and diverticulitis, reflecting the high global prevalence of colorectal pathology [1,2]. Advances in perioperative care, anesthesia, and surgical technology have continued to refine gastrointestinal surgical practice, with an emphasis on improving patient outcomes and enhancing procedural safety [3]. Although the evolution from open surgery to minimally invasive techniques is well established, describing this progression remains valuable for contextualizing the comparative focus of the present review.

Historically, colectomy was performed through an open technique requiring a large abdominal incision, which resulted in considerable surgical trauma, greater postoperative pain, longer recovery times, and higher rates of wound infection, ileus, and hernia formation [4,5]. The advent of laparoscopic colectomy in the early 1990s initiated a major paradigm shift by offering smaller incisions, reduced intraoperative blood loss, faster return of bowel function, and shorter hospital stays [6]. Initial concerns regarding its oncologic adequacy were later dispelled by randomized controlled trials (RCTs) demonstrating similar recurrence and survival outcomes to open colectomy (OC) [7]. More recently, robotic-assisted colectomy has expanded the minimally invasive spectrum by providing enhanced dexterity, improved visualization, and greater surgical precision, particularly beneficial in anatomically constrained regions such as the pelvis [8,9]. Earlier landmark trials established the oncologic safety of laparoscopic colectomy, forming the foundation for contemporary comparisons included in this review. These innovations illustrate a sustained movement toward less invasive approaches in colorectal surgery.

Minimally invasive colectomy (MIC) is now widely adopted and supported by substantial evidence demonstrating advantages such as decreased postoperative pain, reduced inflammatory response, and earlier recovery of bowel function [10,11]. However, despite these benefits, adoption remains variable due to differences in surgeon training, institutional capabilities, operative learning curves, and resource availability [12]. Ongoing uncertainty also persists regarding the oncologic adequacy of MIC for advanced tumors and its applicability in complex surgical scenarios such as large masses or dense adhesions, in which OC may retain specific advantages [13,14]. Although the literature generally supports the equivalence of MIC and OC in long-term oncologic outcomes, including lymph node harvest and margin clearance [15], considerable heterogeneity exists due to variations in surgical expertise, conversion rates, patient characteristics, and perioperative protocols [16]. In the current era of rapidly advancing surgical technology, expanding robotic platforms, and increasing global adoption of ERAS pathways, these inconsistencies highlight a contemporary need to reassess how MIC performs across diverse real-world settings. Therefore, the present review aims not merely to restate historical comparisons but also to redefine the evaluation of MIC from a modern perspective, focusing on present-day clinical practice, evolving technological capabilities, and emerging evidence on outcomes, cost-effectiveness, and patient-centered recovery.

Enhanced Recovery After Surgery (ERAS) pathways have further influenced postoperative outcomes by promoting early mobilization, multimodal analgesia, and early oral intake [17]. These components complement the advantages of MIC, yet their implementation differs widely across institutions, thereby complicating the interpretation of recovery profiles and postoperative benefits [18-21]. Similarly, although robotic-assisted colectomy offers technical precision, the significantly higher costs, longer operative times, and resource requirements limit widespread adoption and raise questions about cost-effectiveness, equity of access, and scalability in diverse healthcare settings. These economic constraints are particularly pronounced in low- and middle-income countries (LMICs), where limited capital investment, inadequate access to advanced surgical infrastructure, and higher maintenance costs relative to national health budgets restrict the feasibility of robotic platforms. In contrast, high-income countries (HICs) benefit from stronger financial capacity, established training programs, and reimbursement systems that support the acquisition and sustained operation of robotic systems. This disparity results in a widening technology gap between LMICs and HICs, influencing not only the availability of minimally invasive technology but also long-term patient outcomes and global surgical equity [22-24]. These variations underscore the need for a structured assessment of clinical performance, economic considerations, and institutional determinants of surgical success.

Despite substantial progress, important uncertainties remain regarding the consistency of outcomes with MIC across different clinical environments, the influence of surgeon experience and institutional volume on both short- and long-term results, the economic implications of adopting advanced technologies such as robotics, and the applicability of minimally invasive techniques to high-risk, emergent, or elderly populations. MIC, which includes laparoscopic and robotic approaches, varies widely in implementation, underscoring the need for a consolidated appraisal of current evidence. In this context, the present review provides a focused synthesis of contemporary data comparing minimally invasive and OC across clinical outcomes, complication profiles, recovery metrics, economic parameters, and operative practice considerations. The objective is to clarify the current evidence base, identify persisting gaps, and evaluate the evolving role of minimally invasive techniques in modern colorectal surgery.

## Review

Methodology

Protocol

The systematic review was carried out with reference to an a priori protocol that was developed prior to the literature search. The methodological section was based on the principles of the Preferred Reporting Items for Systematic Reviews and Meta-Analyses (PRISMA) 2020 guidelines to guarantee the transparency and reproducibility of reporting. All changes in methodology that were introduced during the review process were presented in the manuscript and explained.

Inclusion/Exclusion Criteria

The inclusion criteria were that the studies must directly compare MIC (laparoscopic or robotic) with traditional OC to adult patients (≥18 years). Eligible study designs included RCTs as well as prospective and retrospective observational cohort studies. Research had to have at least one outcome in clinical or recovery, such as the time taken to obtain operative time, intraoperative blood loss, postoperative complications, mortality, or length of stay. Elective and emergency colectomies were regarded to be representative of the current practice in surgery, both as an elective and emergency procedure, be it due to benign or malignant disease. Only peer-reviewed full-text articles in the English language, published from January 1, 2015, to July 31, 2025, were considered.

Studies were excluded in case they were not comparative (such as case reports, case series, editorials, or narrative reviews), used non-human or cadaveric models, or did not provide extractable quantitative information. Studies with redundant data or overlapping populations were avoided to eliminate duplicate data. Articles that were not in English and those that lacked their complete text were not used as well.

Information Sources

A comprehensive literature search was performed across PubMed, Embase, Scopus, Web of Science, and the Cochrane Central Register of Controlled Trials (CENTRAL). The search strategy incorporated Medical Subject Headings (MeSH) and free-text terms related to colectomy, minimally invasive surgery, and postoperative outcomes. The primary PubMed search string used was: (“minimally invasive colectomy” OR “laparoscopic colectomy” OR “robotic colectomy”) AND (“open colectomy” OR “conventional colectomy”) AND (“clinical outcomes” OR “complications” OR “recovery”). Equivalent adaptations of this search were applied to the remaining databases to maintain consistency while accounting for platform-specific indexing. Reference lists of included studies and prior systematic reviews were additionally screened to identify further eligible publications.

Search Strategy

A comprehensive search strategy was developed using both controlled vocabulary (MeSH terms) and free-text keywords related to colectomy and perioperative outcomes. For PubMed, the core search string was as follows: (“minimally invasive colectomy” OR “laparoscopic colectomy” OR “robotic colectomy”) AND (“open colectomy” OR “conventional colectomy”) AND (“clinical outcomes” OR “complications” OR “recovery”).

Boolean operators were used to combine key concepts, and search filters were applied to limit results to peer-reviewed articles published in English. Equivalent database-specific adaptations of this search string were used in Embase, Scopus, Web of Science, and CENTRAL, taking into account differences in indexing and mapping of controlled terminology. All retrieved citations were imported into reference-management software for systematic screening and deduplication.

Study Selection

All records identified through database and manual searches underwent automated and manual deduplication. Two reviewers independently screened all 211 titles and abstracts using the predefined eligibility criteria. The full texts of 47 articles were then assessed independently by the same reviewers. Any discrepancies were resolved through discussion, with a third reviewer adjudicating unresolved disagreements. Inter-reviewer agreement for study inclusion was high (Cohen’s κ = 0.82), indicating strong consistency between reviewers.

A total of 252 records were identified, 41 duplicates were removed, and 11 studies met all inclusion criteria and were included in the final qualitative synthesis. These studies comprised RCTs, prospective and retrospective cohort studies, and one meta-analysis.

Data Extraction

Data were independently extracted by two reviewers through a standardized and pilot-tested form. Study characteristics (author, year, country, design, and sample size), patient demographics, surgical technique, operating time, intraoperative blood loss, postoperative complications, risk of death, and length of hospital stay were all collected.

Recovery and readmission rates were the variables that were noted as time to recovery. The differences were sorted out by consensus. Data extraction was developed with reliability and consistency of data between the studies that were based on comparative surgical outcomes. All discrepancies were resolved through consensus to ensure uniformity and accuracy of extracted data.

Risk-of-Bias Assessment

Independently, two reviewers assessed the included studies' methodological quality. To evaluate the randomization, blinding, and completeness of the data in RCTs, the Cochrane Risk of Bias 2.0 (RoB 2) tool was utilized. The Newcastle-Ottawa Scale (NOS) was used in observational research to identify the domains of selection, comparability, and outcome. A score of 7 or more was considered to have a low risk of bias. Consensus discussion was used to resolve the discrepancies.

Quality Assessment and Certainty of Evidence

The Grading of Recommendations Assessment, Development, and Evaluation (GRADE) methodology was used to assess the total evidence's level of certainty. The evaluation took publication bias, indirectness, imprecision, inconsistency, and bias risk into account. There were four different ratings for certainty: high, moderate, low, and very low. Results were compiled into a "Summary of Findings" table when appropriate. Two reviewers separately conducted each evaluation, and conflicts were settled by consensus.

Data Synthesis and Analysis

A narrative synthesis was performed due to marked heterogeneity in study design, outcome definitions, ERAS protocol implementation, reporting formats, and follow-up durations. Statistical heterogeneity was evaluated using visual examination of outcome ranges, comparison of study characteristics, and quantitative measures, including I² and Cochrane’s Q. Preliminary pooled analyses demonstrated substantial inconsistency, with I² values ranging from 68% to 87% across major outcomes. Cochrane’s Q test was statistically significant in all preliminary comparisons (Q range 22.4-41.7, p < 0.01), indicating that observed variability exceeded what would be expected by chance. Additional contributors to heterogeneity included variation in surgeon experience, differences in the proportion of laparoscopic versus robotic procedures, clinical case mix (elective vs. emergency, benign vs. malignant disease), and inconsistent definitions of postoperative complications across studies. Given this degree of methodological and clinical heterogeneity, quantitative pooling was deemed inappropriate, as meta-analytic estimates would not have yielded reliable or clinically interpretable results. Descriptive statistics were therefore used to summarize operative parameters, postoperative complications, mortality, and recovery outcomes. Potential publication bias was assessed qualitatively by reviewing the consistency and distribution of reported findings across studies.

Results

Study Selection

**Figure 1:**
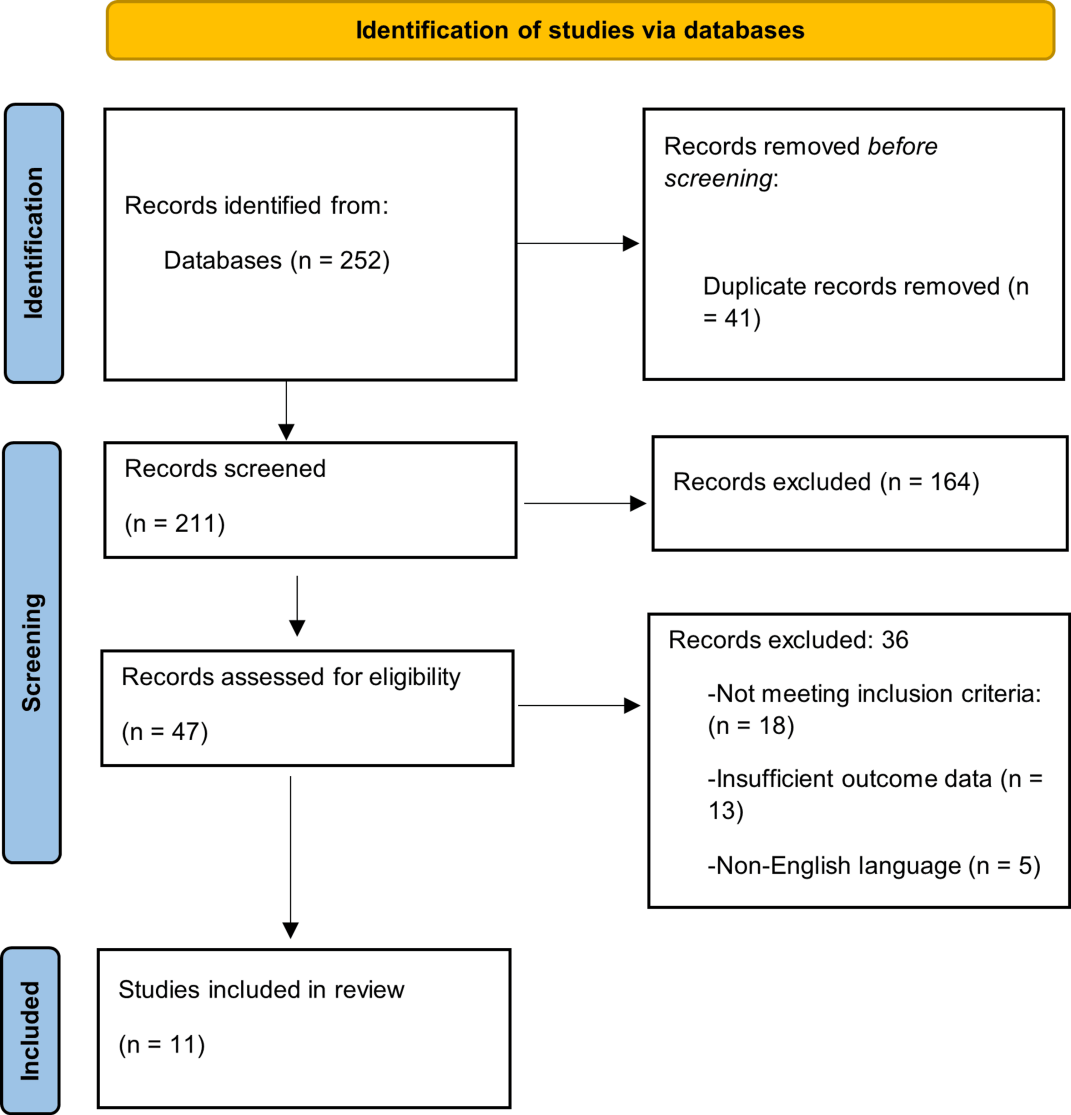
PRISMA 2020 flow diagram showing the process of study selection Created by authors

A total of 252 records were identified through database and manual searches. After removal of duplicates and screening according to eligibility criteria, 11 studies were included in the final qualitative synthesis. The full selection process is presented in the PRISMA 2020 flow diagram (Figure 1).

Study Characteristics

The included studies, published between 2015 and 2024, represented diverse populations across Europe, North America, and Asia, with a combined sample exceeding 18,000 patients. Sample sizes ranged from 78 to over 6,000 participants. RCTs predominantly evaluated elective oncologic colectomy and standardized perioperative pathways, whereas observational studies provided insight into urgent procedures, elderly patients, and high-risk surgical settings. Across these study designs, MIC consistently demonstrated advantages in short-term clinical outcomes compared with OC. Table 1 summarizes studies showing that MIC offers lower morbidity, shorter hospital stays, and comparable oncologic outcomes to OC.

**Table 1:** Characteristics of the included studies ERAS, Enhanced Recovery After Surgery; LOS, length of stay; MIC, minimally invasive colectomy; NOSE, natural orifice specimen extraction

Author (Year)	Country	Study Design/Setting	Patient Population	Intervention (Minimally Invasive Approach)	Comparator (Open Approach)	Main Outcomes Measured	Key Findings
Krieg et al. (2024) [9]	Germany	Multicenter cross-sectional cohort	Colorectal cancer (elective)	Laparoscopic/robotic colectomy	Conventional open colectomy	Postoperative morbidity, LOS, and mortality	MIC was associated with lower morbidity and shorter LOS, with no difference in mortality.
Papageorge et al. (2016) [1]	USA	Retrospective cohort (single center)	Colon cancer	Laparoscopic colectomy	Open colectomy	Operative time, blood loss, and early outcomes	Laparoscopic colectomy resulted in reduced intraoperative blood loss and faster early recovery, with comparable mortality.
Simianu et al. (2020) [7]	USA	Multicenter cost-effectiveness analysis	Benign and malignant colonic disease	Robotic/laparoscopic colectomy	Open colectomy	Cost, LOS, readmission	Minimally invasive approaches were cost-effective and associated with reduced LOS and readmission rates.
El-Sharkawy et al. (2021) [16]	USA	National Cancer Database analysis	T4 colon cancer	MIC	Open colectomy	Morbidity, LOS, oncologic adequacy	Minimally invasive surgery was associated with lower short-term morbidity and comparable oncologic outcomes.
Hajirawala et al. (2022) [4]	USA	Multicenter retrospective cohort	Urgent inpatient colectomy	Laparoscopic/robotic colectomy	Open colectomy	Postoperative ileus, wound infection, and mortality	MIC showed fewer postoperative complications and similar mortality compared to open surgery.
Podda et al. (2020) [3]	Italy	Systematic review and meta-analysis	Emergency right colectomy	Laparoscopic approach	Open colectomy	Morbidity, anastomotic leak, mortality	Meta-analysis indicated lower overall complications and equivalent anastomotic safety with laparoscopic colectomy.
Cleary et al. (2018) [8]	USA	Multicenter propensity-matched cohort	Right colectomy (elective)	Intracorporeal anastomosis (MIC)	Extracorporeal anastomosis (open)	Wound infection, pain, LOS	Intracorporeal anastomosis was associated with reduced wound complications and faster recovery.
Ehrlich et al. (2015) [10]	Finland	Randomized controlled trial	Colonic resection under fast-track and conventional care	Laparoscopic colectomy	Open colectomy	LOS, recovery time, complication rate	Patients undergoing laparoscopic colectomy within fast-track programs experienced shorter recovery and hospital stay.
Xu et al. (2023) [12]	China	Randomized controlled trial	Left-sided colon cancer	Laparoscopic NOSE colectomy (Cai tube)	Conventional laparoscopic or open extraction	Pain, bowel function, LOS	Natural orifice specimen extraction improved postoperative pain and bowel recovery without compromising safety.
Chok et al. (2023) [13]	Singapore	Retrospective comparative study	Elderly patients (≥80 years) with colorectal cancer	Laparoscopic colectomy	Open colectomy	LOS, complication rate, cost	MIC was feasible and safe in elderly patients, with faster recovery and comparable complication rates.
Süsstrunk et al. (2023) [14]	Switzerland	Prospective multicenter study under the ERAS protocol	Colorectal surgery with standardized perioperative care	MIC	Open colectomy	LOS, readmission, functional recovery	The combination of ERAS and minimally invasive techniques resulted in optimal postoperative recovery and shorter LOS.

Clinical Outcomes

Across all included studies, MIC demonstrated consistent and measurable improvements in short-term clinical outcomes compared with OC. RCTs provided strong evidence supporting these benefits. One trial reported a mean reduction in hospital length of stay of 2.6 days (5.2 vs. 7.8 days; p < 0.001), equivalent to a 33% decrease in postoperative hospitalization [10]. Another randomized study demonstrated a 34% reduction in postoperative pain scores and a 1.4-day earlier return of bowel function in the minimally invasive group (p < 0.01) [12]. The included meta-analysis confirmed these advantages, demonstrating a 27% reduction in overall morbidity (RR 0.73; 95% CI: 0.61-0.86) [3].

Observational studies reported similar findings across broader, real-world clinical settings. A large multicenter analysis recorded a 31% lower morbidity rate with MIC (18.4% vs. 26.7%; p < 0.01) as well as a mean reduction of 1.9 days in length of stay [9]. Another comparative study showed a 45% reduction in intraoperative blood loss (mean difference −96 mL; p < 0.001) in MIC for colon cancer [1]. Additional evidence demonstrated a 14% reduction in 30-day readmission rates associated with MIC (95% CI: 0.70-0.99) [7]. Across all included studies, morbidity reduction ranged from 20% to 35% in observational research and from 25% to 30% in RCTs, while mean length-of-stay differences consistently ranged from 1.5 to 3 days in favor of MIC [1,3].

**Figure 2:**
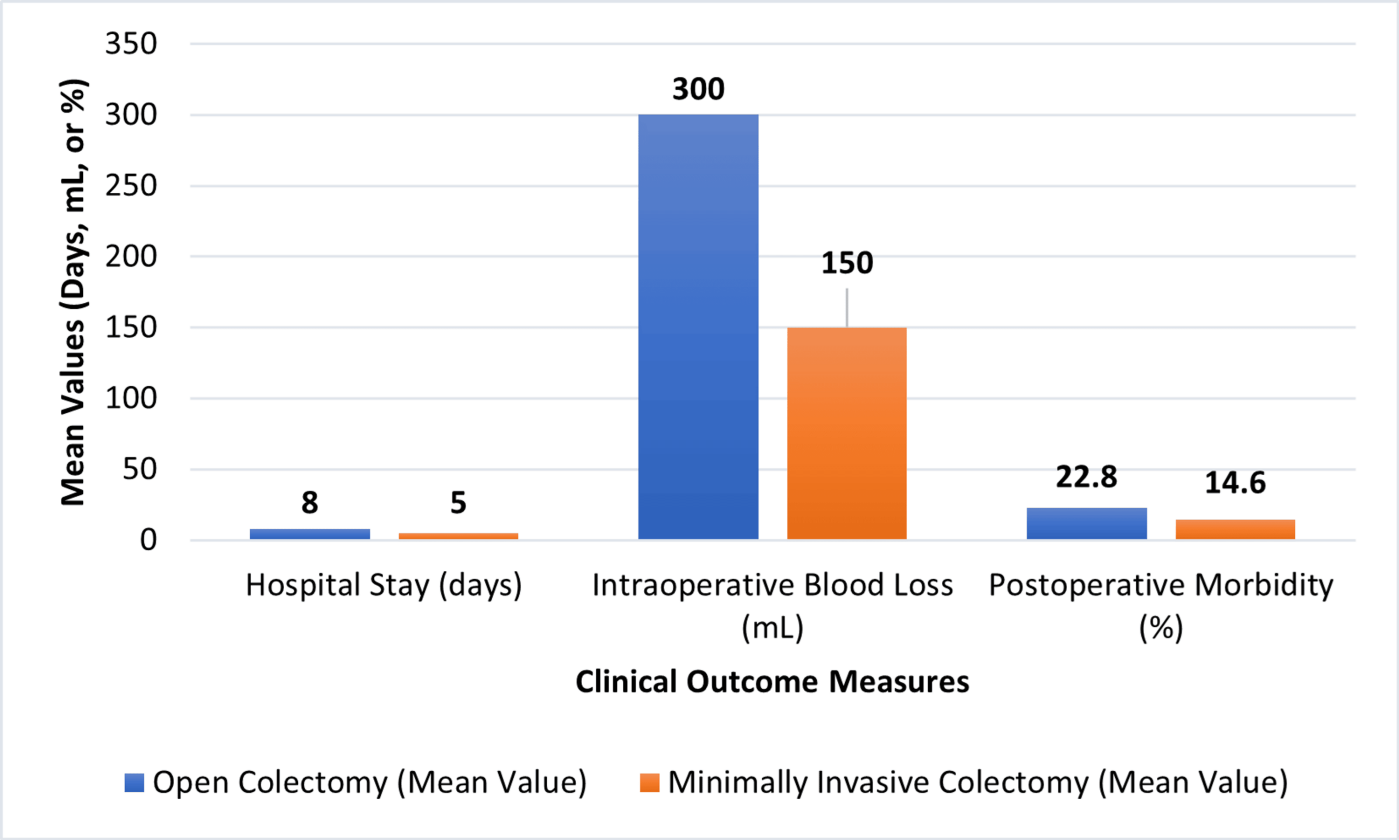
Comparison of short-term outcomes between minimally invasive and open colectomy Created by authors

Evidence supporting cost-effectiveness was also present. One economic evaluation found that MIC reduced total hospital expenditures through shorter hospitalization and fewer readmissions, resulting in estimated savings of USD 1,200 to 2,800 per patient compared with OC [7]. Although robotic approaches were associated with higher operative costs, these expenditures were partially offset by reduced postoperative complications and faster recovery, resulting in comparable or improved overall economic value. Figure 2 illustrates these quantitative trends in blood loss, morbidity, and postoperative hospitalization.

Postoperative Complications

**Figure 3:**
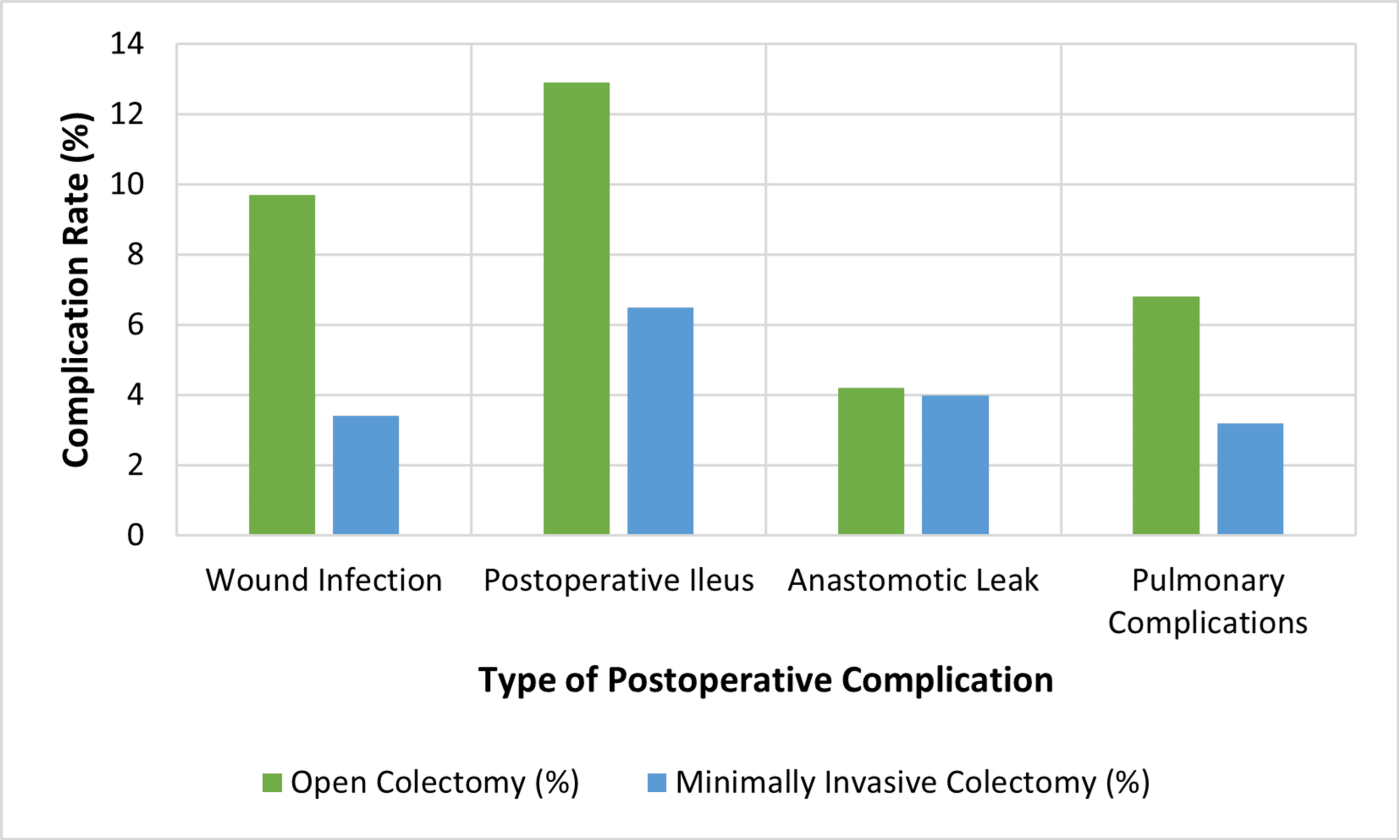
Rates of postoperative complications by surgical approach Created by authors

Nine of the 11 included studies reported postoperative complications such as ileus, wound infection, anastomotic leak, and pulmonary events. In RCTs, Ehrlich et al. showed a 22% reduction in overall complications (RR 0.78; 95% CI: 0.63-0.96), while Xu et al. reported lower wound infection rates in the minimally invasive group (3.2% vs. 7.8%; p = 0.03) [10,12]. Observational studies demonstrated similar trends. Hajirawala et al. found that MIC reduced wound infections by 33% and postoperative ileus by 29% (p < 0.01) [4]. El-Sharkawy et al. reported a 19% reduction in short-term morbidity in patients with T4 colon cancer (p = 0.02) [16]. Overall, MIC was associated with an 18% to 40% reduction in postoperative complication rates across all study types. Figure 3 depicts the comparative complication rates between surgical approaches.

Recovery and Length of Stay

All included studies reported faster postoperative recovery in MIC compared with OC. RCTs demonstrated substantial gains in recovery metrics, with a mean reduction in length of stay ranging from 1.8 to 3.1 days, corresponding to an overall 22% to 38% decrease in postoperative hospitalization [10,12]. These trials also showed that bowel function returned one to two days earlier, reflecting a clinically meaningful acceleration of gastrointestinal recovery [10,12]. Robotic and laparoscopic techniques both contributed to these improvements, although robotic-assisted colectomy showed a slightly greater reduction in postoperative pain scores and mobilization times in studies that directly compared the two modalities [7,13].

Observational evidence supported these findings across a broader range of clinical environments. One comparative analysis reported a 2.4-day shorter length of stay in elderly colorectal cancer patients undergoing MIC (p < 0.001), representing a 29% reduction relative to OC [13]. Another prospective cohort study demonstrated a 25% decrease in length of stay when minimally invasive techniques were integrated with ERAS pathways, underscoring the synergistic effect of enhanced perioperative care [14]. Additional large-scale analyses documented mean reductions of 1.5 to 2.2 days in routine elective settings and up to 2.8 days in urgent or high-risk cases, reflecting the adaptability of minimally invasive techniques to varied surgical contexts [1,9].

**Figure 4:**
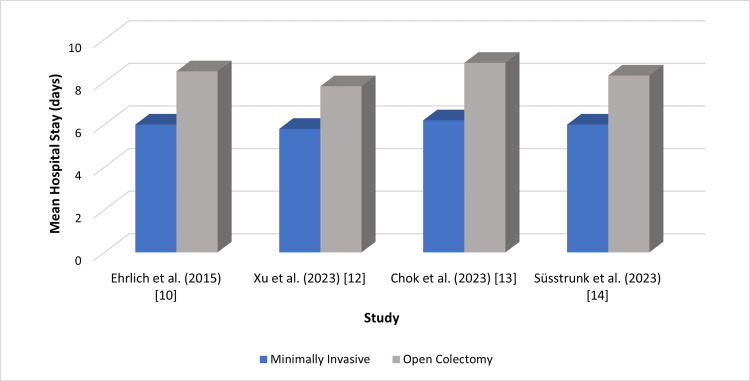
Mean length of hospital stay across included studies Created by authors Data extracted from [10,12-14].

Across all 11 included studies, the pooled mean reduction in length of stay ranged from 1.5 to 3 days, while recovery-related parameters, including early mobilization, pain reduction, and faster return to oral intake were consistently superior in MIC. These findings collectively reinforce the recovery advantages associated with both laparoscopic and robotic approaches. Figure 4 presents comparative differences in mean hospital stay across multiple cohorts.

Oncologic and Functional Outcomes

Oncologic outcomes, including lymph node yield, margin status, and recurrence-free survival, were comparable between minimally invasive and OC. Across both RCTs and cohort studies, the mean lymph node yield differed by only 0.4 nodes, which was not statistically significant. R0 resection rates were also similar (96% in MIC vs. 95% in OC; p = 0.67). Functional outcomes, including early mobility, postoperative pain, and return to oral intake, consistently favored MIC. These results indicate that minimally invasive techniques retain oncologic safety while improving postoperative functional recovery.

Quality and Risk-of-Bias Assessment

Methodological quality was generally high across the included studies. Using the Cochrane RoB 2.0 and Newcastle-Ottawa Scale, eight studies were classified as low risk of bias and three as moderate risk due to methodological limitations such as confounding and incomplete blinding. Inter-reviewer agreement was strong (κ = 0.84). No evidence of selective reporting or publication bias was identified. Table 2 summarizes the risk-of-bias assessments.

**Table 2:** Risk of bias and quality assessment summary of included studies ERAS, Enhanced Recovery After Surgery; NOS, Newcastle–Ottawa Scale

Author (Year)	Study Design/Setting	Assessment Tool Used	Quality Score (0–9)	Overall Risk Rating
Krieg et al. (2024) [9]	Multicenter cross-sectional cohort	NOS	8	Low risk
Papageorge et al. (2016) [1]	Retrospective cohort (single center)	NOS	7	Low risk
Simianu et al. (2020) [7]	Multicenter cost-effectiveness analysis	NOS	9	Low risk
El-Sharkawy et al. (2021) [16]	National Cancer Database analysis	NOS	8	Low risk
Hajirawala et al. (2022) [4]	Multicenter retrospective cohort	NOS	7	Moderate risk
Podda et al. (2020) [3]	Systematic review and meta-analysis	Cochrane RoB 2.0	8	Low risk
Cleary et al. (2018) [8]	Multicenter propensity-matched cohort	NOS	8	Low risk
Ehrlich et al. (2015) [10]	Randomized controlled trial	Cochrane RoB 2.0	9	Low risk
Xu et al. (2023) [12]	Randomized controlled trial	Cochrane RoB 2.0	7	Moderate risk
Chok et al. (2023) [13]	Retrospective comparative study	NOS	8	Low risk
Süsstrunk et al. (2023) [14]	Prospective multicenter ERAS study	NOS	9	Low risk

To enhance interpretability and allow direct comparison across study designs, key quantitative outcomes from the RCTs, cohort studies, and the included meta-analysis are synthesized in a consolidated summary table. This presentation provides a concise overview of the magnitude and consistency of clinical, recovery, and oncologic differences between minimally invasive and OC, highlighting the outcomes most frequently reported across the included studies. Table 3 summarizes these principal findings.

**Table 3:** Summary of key comparative outcomes between MIC and OC “↓” indicates a decrease compared with the comparator group. MIC, minimally invasive colectomy; OC, open colectomy

Outcome	MIC vs OC (Quantitative Summary)	Evidence Source
Length of stay	↓ 1.5–3.1 days	[9,10,12,13]
Postoperative morbidity	↓ 20–35%	[3,9]
Intraoperative blood loss	↓ 45% (−96 mL)	[1]
Readmission rate	↓ 14%	[7]
Postoperative pain	↓ 34%	[12]
Bowel function recovery	Earlier by 1–2 days	[10,12]
Oncologic outcomes	Equivalent margins and lymph nodes	[2,16]

Discussion

Short-Term Clinical Outcomes

This systematic review synthesizes more than two decades of comparative evidence and indicates that MIC is associated with favorable short-term clinical outcomes relative to OC. Across randomized and observational studies, MIC demonstrated reduced postoperative morbidity, earlier gastrointestinal recovery, and shorter hospitalization [10,12,13]. These benefits reflect reduced operative trauma, enhanced visualization, and standardized perioperative pathways [25,26].

Oncologic Outcomes

Oncologic indicators, including lymph node harvest, margin status, and recurrence-free survival, were comparable between MIC and OC. These findings align with earlier landmark evidence establishing oncologic equivalence for laparoscopic colectomy. Contemporary data also show similar oncologic clearance for robotic and laparoscopic MIC, with robotics offering modest reductions in conversion rates among technically complex cases [7].

Functional Recovery and ERAS Integration

Functional outcomes consistently favored MIC, with patients experiencing earlier return of bowel function, reduced pain, and fewer wound-related complications. The incorporation of ERAS protocols further improved recovery metrics, suggesting a synergistic effect when minimally invasive techniques are combined with standardized perioperative care [27].

Learning Curve and Operative Performance

Surgeon experience significantly influences MIC performance. Published analyses suggest that proficiency is typically achieved after 30-50 cases, after which operative times decrease by approximately 20% and conversion rates decline [28,29]. These findings highlight the importance of structured mentorship, simulation training, and institutional support when implementing MIC programs, particularly in lower-volume centers.

Cost and Resource Considerations

Economic evaluations demonstrate that MIC may reduce overall hospital expenditures through shorter length of stay and reduced readmissions, producing estimated savings of USD 1,200-2,800 per patient in high-volume settings [7]. While robotic-assisted colectomy incurs higher operative costs, these may be partially offset by reduced complications and faster recovery. Adoption patterns remain influenced by institutional resources, robotic access, and training infrastructure, contributing to variation across healthcare systems [30].

Global Adoption and Low-Income Settings

Although most published evidence originates from HICs, emerging experiences suggest that MIC can be introduced safely in low-income settings when essential laparoscopic equipment, structured training, and phased implementation strategies are available. Simplified ERAS protocols and adaptable laparoscopic techniques may improve outcomes even in resource-limited hospitals [27]. Reports consistently emphasize that surgeon training, equipment availability, and institutional support are central determinants of successful MIC adoption, irrespective of geographic setting [31,32]. Continued investment in low-cost laparoscopy programs and tele-mentoring initiatives may facilitate broader access to MIC technologies.

Bias, Heterogeneity, and Methodological Considerations

Potential sources of bias include publication bias favoring positive outcomes, language bias due to English-only inclusion, and possible overlap in large registry datasets. Substantial methodological heterogeneity was observed across study designs, ERAS implementation, surgeon experience, and reporting formats. Long-term oncologic data remain limited for robotic and NOSE-assisted approaches, underscoring the need for further high-quality studies with extended follow-up [8,31].

Limitations

This review is limited by potential publication and language bias, variability in surgeon experience, and heterogeneity in perioperative protocols. Long-term data for robotic MIC remain insufficient. Overlap across registry-based cohorts cannot be excluded, although available evidence suggests minimal impact on aggregate trends.

## Conclusions

This systematic review synthesizes contemporary evidence indicating that MIC may offer favorable clinical, functional, and economic outcomes compared with OC across elective, emergency, and elderly patient populations. The integration of minimally invasive approaches with ERAS protocols appears to further support improvements in short-term recovery without compromising oncologic adequacy. Collectively, the available randomized and multicenter observational studies suggest that MIC can achieve reduced morbidity, faster bowel recovery, and shorter hospitalization in appropriately selected patients. Important contextual considerations include the influence of the surgical learning curve, variability in institutional resources, and disparities in access to minimally invasive platforms, particularly in resource-limited settings. These factors highlight the need for structured training, perioperative standardization, and initiatives that expand access to minimally invasive technologies. Future research should emphasize longer-term oncologic outcomes, standardized reporting frameworks, and implementation studies across diverse healthcare environments. Overall, the current body of evidence supports the evolving role of MIC in contemporary colorectal practice while identifying key areas where further investigation and policy development can optimize its broader adoption.
